# DW-MRI as a Biomarker to Compare Therapeutic Outcomes in Radiotherapy Regimens Incorporating Temozolomide or Gemcitabine in Glioblastoma

**DOI:** 10.1371/journal.pone.0035857

**Published:** 2012-04-20

**Authors:** Stefanie Galbán, Benjamin Lemasson, Terence M. Williams, Fei Li, Kevin A. Heist, Timothy D. Johnson, Judith S. Leopold, Thomas L. Chenevert, Theodore S. Lawrence, Alnawaz Rehemtulla, Tom Mikkelsen, Eric C. Holland, Craig J. Galbán, Brian D. Ross

**Affiliations:** 1 Department of Radiation Oncology, University of Michigan, Ann Arbor, Michigan, United States of America; 2 Department of Radiology, University of Michigan, Ann Arbor, Michigan, United States of America; 3 Department of Biostatistics, University of Michigan, Ann Arbor, Michigan, United States of America; 4 Department of Neurosurgery, Hermelin Brain Tumor Center, Henry Ford Health System, Detroit, Michigan, United States of America; 5 Departments of Cancer Biology and Genetics and Neurosurgery, and Brain Tumor Center, Memorial Sloan-Kettering Cancer Center, New York, New York, United States of America; Wayne State University School of Medicine, United States of America

## Abstract

The effectiveness of the radiosensitizer gemcitabine (GEM) was evaluated in a mouse glioma along with the imaging biomarker diffusion-weighted magnetic resonance imaging (DW-MRI) for early detection of treatment effects. A genetically engineered murine GBM model [Ink4a-Arf^−/−^ Pten^loxP/loxP^/Ntv-a RCAS/PDGF(+)/Cre(+)] was treated with gemcitabine (GEM), temozolomide (TMZ) +/− ionizing radiation (IR). Therapeutic efficacy was quantified by contrast-enhanced MRI and DW-MRI for growth rate and tumor cellularity, respectively. Mice treated with GEM, TMZ and radiation showed a significant reduction in growth rates as early as three days post-treatment initiation. Both combination treatments (GEM/IR and TMZ/IR) resulted in improved survival over single therapies. Tumor diffusion values increased prior to detectable changes in tumor volume growth rates following administration of therapies. Concomitant GEM/IR and TMZ/IR was active and well tolerated in this GBM model and similarly prolonged median survival of tumor bearing mice. DW-MRI provided early changes to radiosensitization treatment warranting evaluation of this imaging biomarker in clinical trials.

## Introduction

Approximately 50% of all patients diagnosed with brain tumors have the most malignant form, glioblastoma multiforme (GBM). Despite aggressive treatments that consist primarily of surgical resection followed by chemoradiotherapy the prognosis remains poor with a median survival of 14 months from diagnosis [1]. The standard of care for glioma patients continues to be concurrent temozolomide and radiotherapy which provides a modest improvement in survival over radiation alone [2]. With a better understanding of the genetic make-up of GBM [3], molecular and genetic profiling is being investigated for biomarkers to predict treatment efficacy [4]. One prognostic factor identified as a reliable biomarker for GBM sensitivity to temozolomide is the methylation status of O^6^-methylguanine-methyl-transferase (*MGMT*) [5]. In a multisite trial, patients with active *MGMT*, an enzyme responsible for DNA repair, were found to receive little benefit from treatment by alkylating agents (i.e. Temozolomide) [6], [7]. Thus new chemotherapeutic drugs are being investigated in the clinic for patients who will unlikely benefit from temozolomide. In this regard Gemcitabine is considered a possible candidate due to its different mechanism of action as it is known to irreversibly inhibit the production of nucleic acids. Emerging results have shown initial promise for use of Gemcitabine as an alternative radiosensitizer for tumors identified as *MGMT* active (unmethylated) [8], [9].

Recent advances to better understand and treat GBMs have also been made by examining alterations in gene amplifications or gene expression by several groups. The Cancer Genome atlas network (TCGA) has cataloged recurrent genomic abnormalities in GBM and has classified GBM based on abnormalities in the genes encoding PDGFRA, IDH1, EGFR and NF1 GBM into four subgroups: the proneural, neural, classical and mesenchymal, respectively [3]. The responses to aggressive therapy have been found to differ by subtype thus this new classification scheme will likely provide a future framework for targeted therapy selection. However, although genetic and molecular biomarkers are proving beneficial at identifying treatment options most likely to succeed [4], they are subject to tumor heterogeneity and once therapy has begun, assessment of response is based primarily on changes in contrast-enhancing tumor volume. The MacDonald criteria for assessing tumor response to treatment are predominantly based on monitoring changes in summed tumor area as measured by CT or MRI 10–12 weeks post-treatment initiation [10]. This approach has been the mainstay of clinical management of glioma patients for the past 20 years. In 2010, the Response Assessment in Neuro-Oncology (RANO) Working Group set new guidelines for assessing therapeutic response that address some of the deficiencies in the MacDonald criteria [11]. While an improvement over its predecessor, RANO continues to assess tumor response by anatomical MRI following the completion of therapy. Thus, while a significant need for improved therapies for the treatment of GBM patients with active MGMT status remains, there also exists the need for development of additional biomarkers of treatment response which could be used to provide an early indication of therapeutic outcome.

Quantitative imaging techniques, derived from positron emission tomography or MRI, are being investigated extensively as biomarkers of tumor response to therapy [12], [13], [14], [15]. The rationale for employing these methodologies is their ability to quantify physiological alterations within the tumor during therapy which may serve as surrogates for overall survival. Diffusion-weighted (DW-) MRI has been studied extensively for its prognostic capabilities in identifying patients responsive to treatment [16], [17]. Treatment-induced loss of tumor cellularity leads to an increase in water mobility that is detectable by DW-MRI since alterations in tumor tissue architecture (such as cell membrane, extracellular matrix and organelles) which restrict the thermal driven displacement of water molecules are reduced [18]. First demonstrated as a biomarker of therapeutic response in 9L glioma-bearing rats treated with a chemotherapeutic (BCNU) [19], [20], DW-MRI has been investigated in clinical studies by many researchers over a variety of tumor types [21], [22], [23], [24].

Due to the complex, and sometime unpredictable, interaction between novel therapeutic agents and glioma biology, various mouse models of GBM have been developed and are currently available to the research community [25], [26], [27]. One animal model wherein key signaling pathways can be turned on and off to investigate targeted therapy is based on the RCAS-tva technology [27], [28]. In an effort to represent the proneural, PDGF driven subtype of human GBM, this mouse model is also PDGF driven where PTEN is deleted in nestin expressing cells in an ink4/arf deficient background [29], [30], [31], [32]. This PDGF driven highly proliferative mouse model has been found to exhibit pathological features similar to the human GBM subtype [30], [33], [34]. Herein we sought to investigate the effectiveness of DW-MRI as a surrogate biomarker of treatment response in this animal model that mimics the proneural GBM class of tumors. Since clinical studies have validated the effectiveness of DW-MRI as an imaging biomarker in glioma patients treated with the temozolomide and radiotherapy [35], [36], [37], [38], it is important to evaluate this biomarker in a preclinical setting exploring the efficacy of promising alternative therapeutic agents (i.e. gemcitabine).

The PDGF-driven genetically engineered model has been shown to express high levels of MGMT in the stem-like GBM cells [39] while the bulk tumor had about a 3-fold lower level of MGMT expression. The lack of epigenetic silencing of the *MGMT* gene in a subset of GBM patients allows for more efficient repair of DNA damage induced by alkylation following treatment with temozolomide chemoradiotherapy. Therefore, there is a clinical need to not only improve radiosensitization of GBM's but also to identify predictive imaging biomarkers of response. Here we demonstrate that gemcitabine, which has been shown to pass the blood-tumor barrier in GBM patients [40], is an excellent radiosensitizer for the proneural PDGF driven GBM subtype, which is in accordance with other pre-clinical data of U251 human glioblastoma cell line treated with GEM/IR [41]. These results support the clinical exploration of gemcitabine in combination with IR as an alternative treatment for GBM patients who fail to respond to TMZ/IR, and whose tumors fall in the proneural PDGF-driven classification.

## Results

### Study 1: Evaluation of Combination TMZ/IR in reducing tumor burden in PDGF-driven mouse GBM

**Figure 1:**
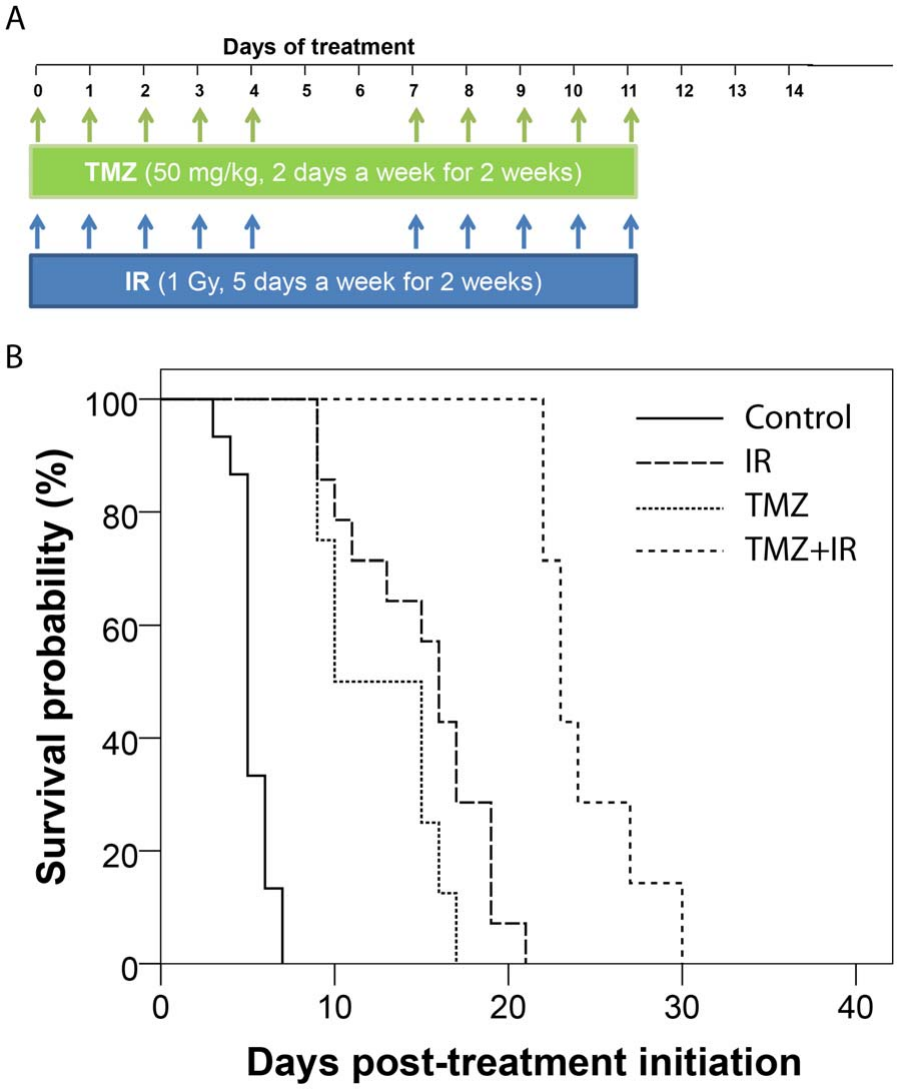
Kaplan-Meier survival plots are presented for each therapy in *Study 1*. (A) Schematic of treatment schedule for *study 1*. Animals were randomized into four groups: control [DMSO/saline], irradiation (IR) [DMSO/saline followed by 1 Gy with 3 hour lag in between treatments], TMZ [50 mg/kg in DMSO/saline] and TMZ+IR [50 mg/kg in DMSO/saline followed by 1 Gy with a 3 hour lag time between treatments]. All treatments were administered five days a week for two weeks.(B) Treatment groups are Controls, irradiation (IR), temozolomide (TMZ) and combination temozolomide and irradiation (TMZ+IR).

As presented in Figure 1, single therapies were significantly more efficacious than vehicle. Median survival was 2 and 3 fold greater for TMZ (10 days (d): 95% confidence interval (CI) 5.8–14.1 d; p<0.0001) and IR (16 d: 14.2–17.8 d; p<0.0001), respectively, than controls (5 d: 4.6–5.4 d). No significant differences in survival were observed between single agent treated groups (p = 0.08). By contrast, combination of TMZ and IR was significantly more efficacious than all other treatment groups (p<0.0001) with a median survival of 23 days (CI = 21.7–24.3 d) and was well tolerated in this GBM model. The body weight loss during treatment never exceeded 10% for all treatment arms. The endpoint of survival was defined as the time point in which the animal had to be removed from the study due to poor health caused by excessive tumor burden.

**Figure 2:**
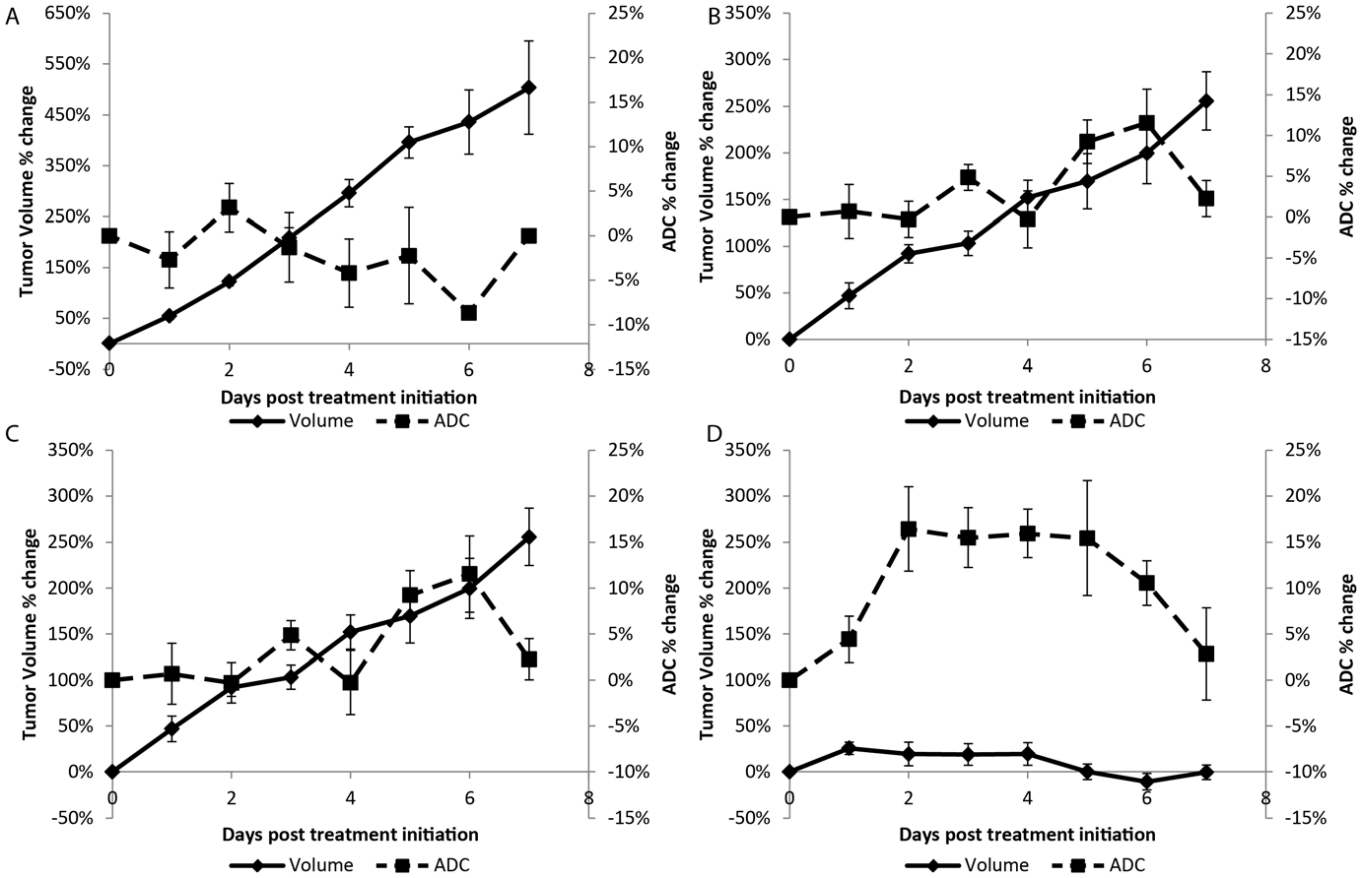
Plots of the percent change in tumor volume and normalized ADC (ADC) for each of the treatment groups in *Study 1*. Presented are treatment groups (A) Control, (B) irradiation (IR), (C) temozolomide (TMZ) and (D) combination temozolomide and irradiation (TMZ+IR). Data is presented over the first week of the study as the mean ± SEM.

The percent change in tumor volume and mean ADC values are presented in Figure 2 for each treatment group over the first week post-treatment initiation. Animals treated with vehicle generated the highest change in tumor volume (doubling time of 2±0.1 days) with negligible percent change in tumor ADC (Figure 2A). In contrast, single agent therapy using IR (Figure 2B) or temozolomide (Figure 2C) and combination therapies (Figure 2D) resulted in a lower volume percent tumor volume change such that significant differences from control were observed as early as two days post-treatment initiation. Although tumor doubling times were extended for single agent therapies (IR: 6±3 days and TMZ: 5±0.4 days) over controls, only chemoradiotherapy was found to completely control tumor growth with tumor volumes at the end of therapy (i.e. 2 week of treatment) at pre-treatment levels that were 3–6 times smaller than tumors treated with single agents (data not shown). Similarities in efficacy for TMZ and IR were observed, nevertheless IR was found to have a more immediate effect on the tumor with ADC percent changes significantly higher than controls by day 1 (Fig. 2B). This suggests substantial cell kill early in IR therapy. ADC values were found to increase steadily throughout TMZ treatment signifying some cell kill, which explains the slow but steady increase in tumor volume (Fig. 2C). A steady increase and decrease was observed for tumor volume and ADC, respectively, following the day 3 of treatment in the IR group suggesting recovery of the tumor from therapy. This was not observed in TMZ-treated animals possibly attributable to residual TMZ in the blood stream. The combination of TMZ and IR was the only therapy capable of controlling tumor growth that even resulted in a drop in tumor volume below pre-treatment values. Following one full cycle of treatment (day 4) chemoradiation resulted in significantly higher changes in tumor mean ADC (Fig. 2D) compared to other treatment groups with mean ADC values due to TMZ+IR treatment increased from baseline by 16% on day 4.

**Figure 3:**
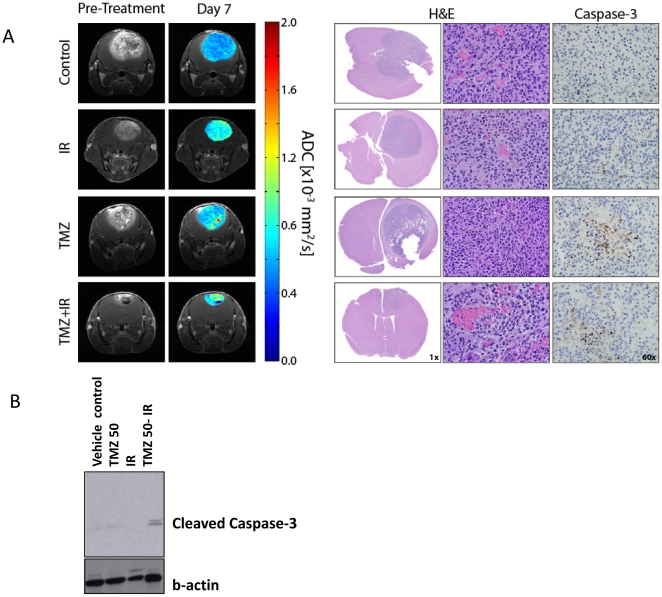
MR, histological images and western blots are presented from representative animals in *Study 1* treatment groups. (A) MRI data consists of anatomical contrast-enhancing T1-weighted images and ADC maps. Histological stains provide information on tumor cellularity (H&E) and apoptosis (cleaved Caspase-3). All data were acquired at day 7 post-treatment initiation. (B) Representative western blot for the detection of cleaved Caspase 3 in tumor tissue from all treatment groups. B-Actin was used as a loading control to ensure proper loading of the protein samples. The tumor tissue from all groups was acquired at day 2 post-treatment initiation.

Depicted in Figure 3A are coronal MR scans and histological sections from representative animals in each treatment group. Two days following the end of the first cycle of vehicle treatment (day 7) low ADC values were observed. These values correlate with negligible caspase-3 staining, a marker for cell apoptosis. In contrast, animals treated with IR, TMZ alone or TMZ/IR generated elevated values in ADC above control and showed positive stained apoptotic cells. Therapeutic response was very spatially heterogeneous in treated animals, partly attributed to spontaneous necrosis and pooling of blood as depicted as hypointense and low ADC (<0.4 mm^2^/s) regions in the CE-T1-weighted images and ADC maps, respectively. The lack of cellularity differences as determined by H&E between groups is attributed to the aggressive nature of this glioma model. As determined from the growth pattern of tumors in vehicle treated animals, the mean doubling time was found to be 42±3 hours.

Induction of cell death in the various treatment groups was further evaluated by conventional western blotting for cleaved caspase-3. As depicted in Figure 3B, tumor tissue at Day two post-treatment initiation from all experimental groups were evaluated for the cleavage of pro-caspase-3 into the two isoforms (17 and 19 kDa). As predicted by DW-MRI elevated levels of the two cleaved caspase-3 forms were detected predominantly in the tumor tissues excised from animals treated with the combination of TMZ and IR indicating an increased level of apoptosis.

### Study 2: Evaluation of Combination GEM/IR in reducing tumor burden in PDGF-driven mouse GBM

**Figure 4:**
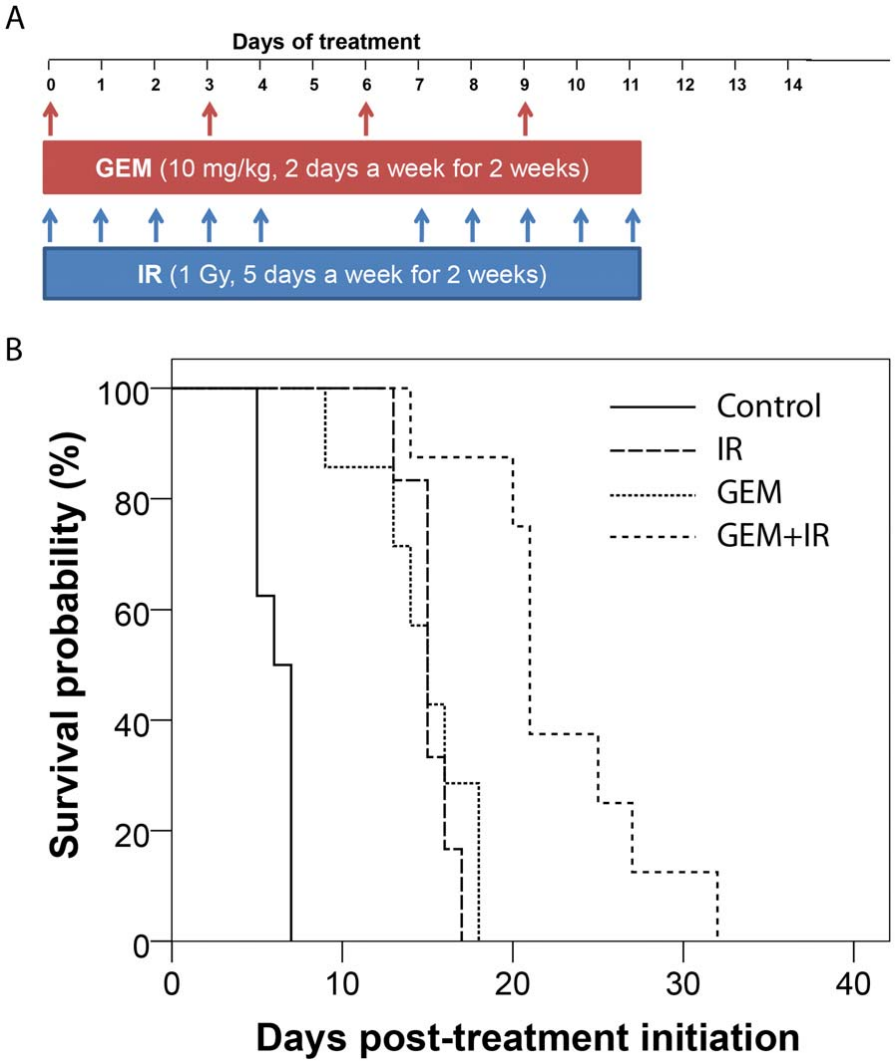
Treatment schedule and Kaplan-Meier survival plots are presented for each therapy in *Study 2*. (A) Treatment schedule schematic for *Study 2*. Animals were randomized into four groups: control, IR, GEM and GEM+IR. Animals of the control group received vehicle 2 days a week for 2 weeks. Animals in the IR group received 1 Gy for 5 days as week with a two day break between treatment blocks for 2 weeks. The GEM group received 10 mg/kg GEM in saline i.p., and GEM+IR received GEM i.p. followed by 1 Gy with a 3 hour lag time between treatments. Control vehicle and GEM administration occurred every third day for a total of four doses. Arrows indicate the day of treatment. (B) Treatment groups are Controls, irradiation (IR), gemcitabine (GEM) and combination gemcitabine and irradiation (GEM+IR).

Single agent treatment with GEM resulted in similar survival plots (Fig. 4) to those observed in *Study 1*. Animals treated with IR or GEM lived significantly longer than control animals with median survival of 15 days for both (IR, CI: 13.5–16.5 d and GEM, CI: 12.4–17.6 d). Combining these therapies improved the median survival to 21 days (CI: 20.1–21.9 d), which was significantly longer than all of the other treatment groups in this study (p<0.05) and was well tolerated. The body weight loss during treatment never exceeded 10% for all treatment arms. The endpoint of survival was defined as the time point in which the animal had to be removed from the study due to poor health caused by excessive tumor burden.

**Figure 5:**
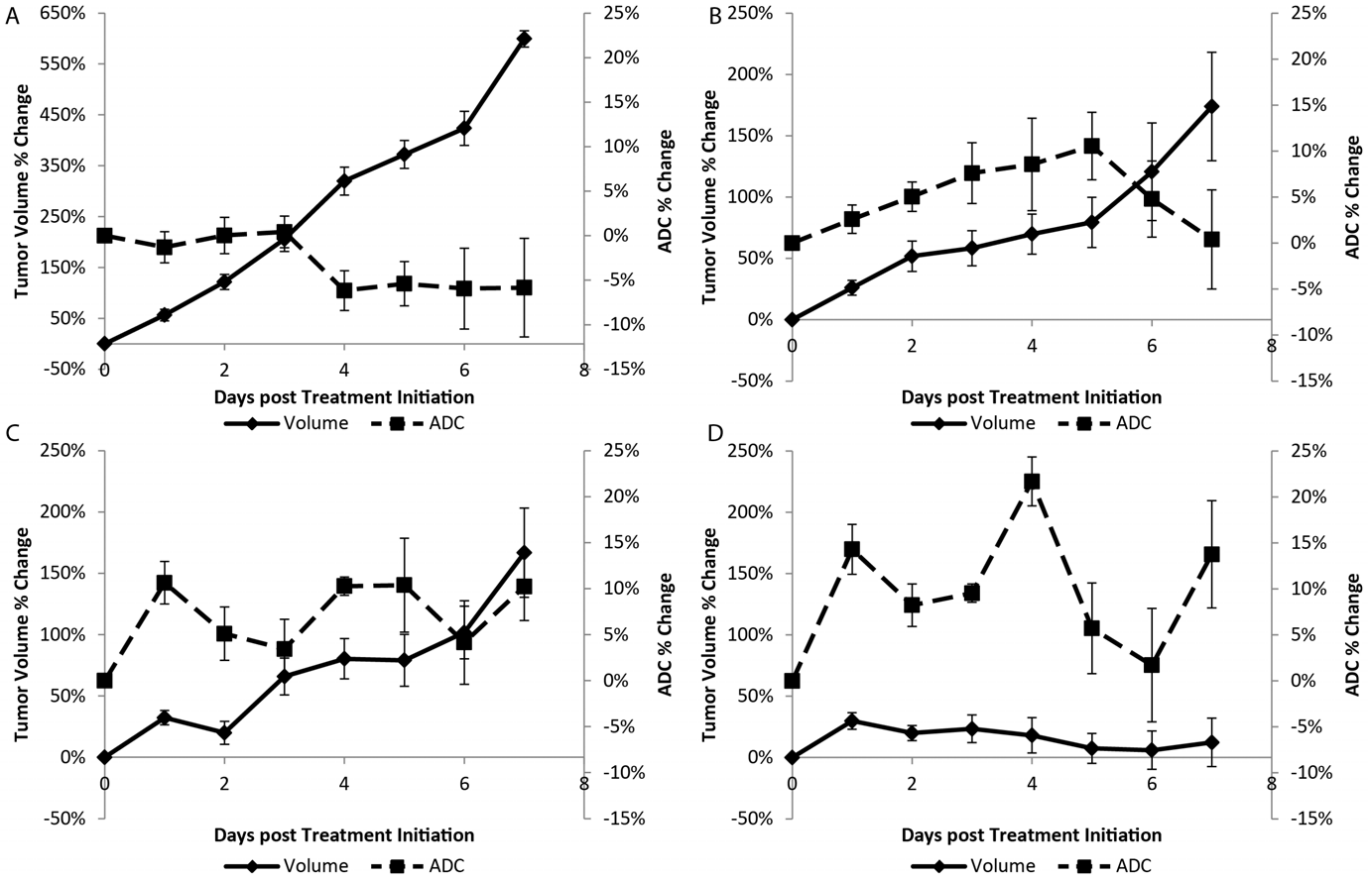
Plots of the percent change in tumor volume and normalized ADC (ADC) for each of the treatment groups in *Study 2*. Presented are treatment groups (A) Control, (B) irradiation (IR), (C) gemcitabine (GEM) and (D) combination gemcitabine and irradiation (GEM+IR). Data is presented over the first week of the study as the mean ± SEM.

Control and IR groups generated profiles in percent change in tumor volume and mean ADC over the first 7 days similar to that observed in *Study 1*, though the vehicle was different. As seen in Figure 5, all treatment groups had significantly lower percent change in tumor volumes from control (doubling time of 2±0.1 days) at day 1 post-treatment initiation. As in *Study 1*, single agent therapies extended tumor doubling times (7±1 days for both IR and GEM) with chemoradiotherapy completely controlling tumor growth during the 2 week cycle. Again, tumor volumes treated with chemoradiation were 4–6 times smaller than their single agent counterparts. IR animals produced ADC percent change values significantly higher than controls by day 4. What is striking about the use of GEM is that although the tumor volume plots behaved similarly to TMZ treatment, the ADC response was almost immediate with over a 10% increase for both single and combination GEM therapies by 24 hours, Figure 5C and Figure 5D, respectively which were significantly higher than controls. In fact, the peak in ADC at days 1, 4 and 7 corresponded to GEM doses delivered 24 hours previously (Fig. 5C, 5D). This sharp increase in ADC suggests massive cell kill in the tumor following the GEM dose. By 48 hours, ADC values immediately decreased towards pre-treatment values, indicating the aggressiveness of this glioma model and its ability to recover from GEM treatment. Inclusion of IR with GEM had a significant effect on the tumors as observed not only in their tumor volume measurements but also in their mean ADC values. Tumor mean ADC values were found to increase by up to 22% from baseline for GEM+IR treatment group by day 4 post-treatment initiation, which was significantly higher than all other therapies (p<0.05).

**Figure 6:**
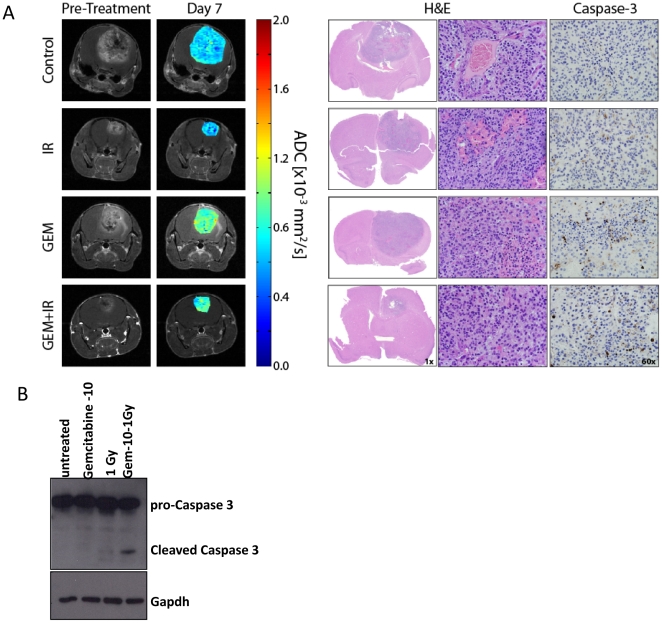
MR and histological images and western blots are presented from representative animals in *Study 2* treatment groups. (A) MRI data consists of anatomical contrast-enhancing T1-weighted images and ADC maps. Histological stains provide information on tumor cellularity (H&E) and apoptosis (caspase-3). All data were acquired at day 7 post-treatment initiation. (B) Tumor tissue from animals left untreated or treated with GEM, IR and GEM+IR at day two post-treatment initiation was assessed for cleaved Caspase 3. Western blot of representative animal tissue is shown and proper loading of protein samples was ensured by probing for Gapdh.

The representative MR images in Figure 6A show elevated ADC values in animals treated with GEM either alone or in combination. These elevated ADC values are a result of the third dose of GEM, which was administered on day 6. Although tumor cellularity between treatment groups as assessed by visual inspection of histology was similar, caspase-3 staining was slightly more pronounced in GEM treated animals as compared to control and IR groups.

Similar to Study 1, proteins from tumor tissues of all treatment groups were harvested and assessed for the induction of apoptosis by cleaved caspase-3 staining. As depicted in Figure 6B the combination of gemcitabine with radiotherapy resulted in an increase of cleaved caspase-3 staining compared to vehicle, GEM, or IR treated animals.

## Discussion

Evaluation of novel or existing therapies or treatment paradigms for GBM can be facilitated by the use of pre-clinical mouse models [42]. Herein we utilized an existing PDGF-driven GBM mouse model wherein known dominant signaling pathways are deregulated, thereby recapitulating the human disease [30], [32]. Specifically, we wanted to evaluate an existing prognostic DW-MRI biomarker (ADC) and demonstrate its utility in predicting treatment outcome in a pre-clinical model, which would allow for assessing drug efficacy in a high throughput format. The existing standard care for GBM patients today is temozolomide in combination with radiotherapy [43]. The addition of temozolomide to radiation alone in the treatment paradigm for GBM patients resulted in a median survival benefit of 2.5 months thus gaining acceptance as standard of care [2]. However compared to other cytotoxic agents, temozolomide is a relatively poor radiosensitizer and the results of a recent multisite study have demonstrated that patients with unmethylated *MGMT* gene promoter will benefit relatively little from this treatment [5], [6], [8]. Methylation of the *MGMT* promoter occurs in about 30 to 60% of glioblastoma patients which is associated with favorable patient outcome using alkylating agents [44], [45]. This and other findings have prompted the pre-clinical investigation and several clinical trials to consider combination therapy of gemcitabine with radiation in GBMs [9], [40]. Gemcitabine, unlike temozolomide is an excellent radiosensitizer as demonstrated by numerous studies both *in vitro* and an *in vivo* [8], [40], [44] and exerts its antitumor effects independent of MGMT status [9]. Gemcitabine's potential as a radiosensitizer was first demonstrated in the human glioblastoma cells U251, which were radiosensitized by a nontoxic concentration of 10 nM [41]. In a different study ectopic human deoxycytidine kinase gene expression was shown to enhance the cytotoxic and radiosensitizing effect of gemcitabine on experimental C6 and U373 intracranial gliomas [35]. Only a few clinical studies have thus far been performed with gemcitabine in the GBM patient population [9], [40], [44]. In a phase 0 study, gemcitabine was found to cross the blood-tumor barrier [40] and in a Phase II study gemcitabine followed by radiotherapy was determined to be a tolerable and safe treatment for newly diagnosed GBM patients yet the GEM schedule used did not confer a survival benefit [46]. However, promising results were obtained in a phase I dose finding study with a fixed dose-rate of GEM (175 mg/m^2^/weekly) [47].

The current study consisted of two Studies wherein DW-MRI was tested as a prognostic biomarker. Study 1 evaluated DW-MRI as a response metric to the glioma model treated with temozolomide and radiation therapy, the standard of care. Performing the same analysis as in Study 1, Study 2 evaluated DW-MRI using gemcitabine, a promising alternative therapy, concurrent with radiotherapy. As designed, single therapies resulted in improved survival over control. For both Study 1 and Study 2, IR treated animals exhibited a sharp increase in ADC suggesting a sufficient drop in tumor cellularity resulting from cell kill. Following treatment (day 4) ADC values regressed back towards baseline. TMZ treated animals did not generate the profile in ADC observed for IR. In fact, ADC values remained elevated even after treatment had ceased on day 4. GEM yielded the most unique ADC profile. The response of the tumor to the drug as determined by ADC was almost immediate. It is highly likely that the peak ADC value was not obtained due to the insufficient temporal resolution for such an aggressive tumor. When combining therapies the ADC profile was found to generate changes in values significantly higher than what was observed for single agent treatments. In the case of Study 1, the profile of ADC over time was similar between IR and TMZ+IR therapies. Contrary to Study 1, the effect of GEM was clearly evident in the ADC profile for both GEM and GEM+IR therapies.

The results of this study showed that changes in ADC were sensitive to tumor response to treatment but were unable to definitively predict the efficacy between treatment groups. As validated by the rapid change in ADC following treatment and the apparent lack of cellularity differences between groups by histology, the aggressiveness of this particular tumor model likely contributes to an attenuated ADC measurement. As observed in the contrast-enhancing MR images in Figures 3 and 6, there was a high prevalence in hypo-intense regions within the tumor that added additional challenges when using this model. These regions were partly due to spontaneous necrosis and blood pooling and had to be filtered prior to ADC analysis as these regions are difficult to accurately quantify by DW-MRI due to the lack of signal. In addition, this glioma model was found to be highly sensitive to chemoradiotherapy, which required daily monitoring of tumor volume and ADC measurements to get an accurate profile of tumor response. When treating with combination therapy, tumor volumes decreased rapidly and reached volumes below the pre-treatment value. This made it difficult to achieve accurate tumor volume and ADC values. Finally, tumor delineation was performed by contouring on the enhancing rim of the tumor. Additional MR modalities (i.e. T2 weighted and FLAIR) were tested but none provided the needed contrast to identify the tumor margins. As a consequence, the contrast-enhancing rim may have extended into healthy brain tissue introducing error into tumor volume measurements.

Diffusion MRI changes are usually indicative of changes in tumor cellularity caused by cell death. Thus tumor tissue from all treatment groups was evaluated by histology and western blotting. In general, positive caspase-3 staining was identified in all chemotherapeutic treatment groups, yet was most pronounced in the combination groups. While large differences in tumor volume were observed between groups at day 7 post-treatment initiation, at the same time point there was an apparent lack of cellularity differences between the groups as analyzed by H&E staining. A possible explanation of this discrepancy could be the aggressive nature and high proliferation rate of this particular glioma model which would allow the tumor cellularity to recover quickly following treatment. This would be in agreement to the sudden drop in ADC values observed at day 7 following IR and 24 hours after each successive GEM dose.

As defined by Verhaak et al.'s classification [3] the major features of the proneural GBM class apart from PDGFRA alterations were point mutations in IDH1. The mouse model used in this study was IDH1 and IDH2 wild-type, thus our findings revealed that combining gemcitabine with radiation was efficacious in reducing tumor burden and prolonging median survival when compared to radiation or gemcitabine alone in this class of GBM. Therefore, this study demonstrated the efficacy of combining gemcitabine with radiotherapy as an alternative treatment strategy for GBMs of the proneural subtype and the utility of DW-MRI as a prognostic imaging biomarker shown to be capable of early quantification of treatment outcome. The design of future GBM clinical trials should include the use of the DW-MRI biomarker in order to provide additional metrics for quantitative and spatial assessment of tumor responsiveness.

## Materials and Methods

### Cell culture

DF-1 cells were purchased from ATCC. Cells were grown at 39°C according to ATCC instructions. RCAS-PDGF-B-HA or RCAS-Cre were a gift from E. Holland and have been described previously [29], [30], [36], [38]. Transfections with RCAS-PDGF-B-HA or RCAS-Cre were performed using Fugene 6 transfection kit according to manufacturer instructions (Roche Roche Applied Science, Indianapolis). Expression of PDGF and Cre was confirmed by western blotting (HA-HRP antibody (Sigma, St. Louis, MO and Cre (Covance Inc., USA).

### Intracranial inoculation

All animal work was conducted according to University of Michigan Laboratory of Animal Management Guidelines under UCUCA Protocol#09583.The University of Michigan Laboratory Animal Committee approved of the use of animals for this study. Generation of the Nestin-tv-a, ink4a-Arf^−/−^/, Pten^loxp/loxp^ mouse line s have previously been described [29], [30], [36], [37], [38].. The animals were originally acquired from E. Holland and inbred at the University of Michigan ULAM facility. 4–6 week old transgenic mice (Nestin-tv-a, ink4a-Arf^−/−^/, Pten^loxp/loxp^) were anesthetized with ketamine (0.1 mg/kg) and xylazine (0.02 mg/kg). One microliter of 8×10^4^ cell mixture containing an equal amount of RCAS-PDGF-B and RCAS-Cre transfected DF1 cells was delivered using a 30-gauge needle attached to a Hamilton syringe and stereotactic fixation device (Stoelting, Wood Dale, IL). Cells were injected to the right frontal cortex: coordinates bregma 1.5 mm, Lat 0.5 mm, and a depth 1.5 mm. Mice were monitored carefully and sacrificed when they displayed lethargy or head tilt due to tumor burden. Institutional (UCUCA and ULAM) guidelines were used to assess the degree of morbidity by following endstage-illnesse scoring procedure and a tumor burden scoring system. Animals remained in the study until they became moribund. If animals displayed severe signs of morbididty, or a moribund state animals were were graded by appearance, natural behavior, provoked behavior, and body condition score.

### Treatment

Following intracranial inoculation tumor volumes were monitored and calculated by contrast-enhanced T1-weighted MRI imaging as described below. Once tumor volume reached 20–40 mm^3^ pre-treatment MRI images were acquired and treatment was initiated. The animals were randomized into 4 different treatment groups per study (n≥8 per group). Temozolomide (TMZ) was prepared in a mixture of 60% dimethyl sulfoxide (DMSO) and 40% saline. Gemcitabine (GEM) was prepared by dissolving in saline. Solutions were prepared fresh and administered within one hour of preparation. Therapeutic agents were purchased from LKT laboratories, Inc., St. Paul, MN, USA.

For cranial irradiation (IR), mice were restrained in a small plastic restraining device and the area to be irradiated (whole brain) was exposed while the rest of the body was shielded with lead to decrease radiation toxicity to normal tissues.

Based on preliminary dose finding experiments, we identified GEM (10 mg/kg), TMZ (50 mg/kg) and 1 Gy IR as minimally efficacious doses at multiple doses instead of one weekly dose and the schedule combination was chosen for our GBM mouse model to yield enhanced survival in combination with a radiosensitizer such as GEM.

#### Study 1

Animals selected for this study were randomized into four groups: control, irradiation (IR), TMZ and TMZ+IR. Treatments were administered as followed: Control animals received an intraperitoneal injection of DMSO/saline, the IR group received DMSO/saline i.p. followed by 1 Gy with 3 hour lag time between treatments, the TMZ group received 50 mg/kg TMZ in DMSO/saline i.p., and the TMZ+IR group received TMZ i.p. followed by 1 Gy with a 3 hour lag time between treatments. All treatments were administered five days a week for two weeks.

#### Study 2

Animals selected for this study were randomized into four groups: control, IR, GEM and GEM+IR. Treatments were administered as followed: Control animals received an intraperitoneal injection of saline, the IR group received saline i.p. followed by 1 Gy with 3 hour lag time between treatments, the GEM group received 10 mg/kg GEM in saline i.p., and GEM+IR received GEM i.p. followed by 1 Gy with a 3 hour lag time between treatments. Control vehicle and GEM administration occurred every third day for a total of four doses. IR was administered as described in Study 1.

### MRI Scans

MRI scans were performed on a 9.4T, 16 cm horizontal bore (Agilent Technologies, Inc., Santa Clara, CA) *Direct Drive* system with a mouse head quadrature volume coil or mouse surface receive coil (m2m Imaging, Corp., Cleveland, OH) actively decoupled to a whole-body volume transmit coil (Rapid MR International, LLC., Columbus, OH). Throughout the MRI experiments, animals were anesthetized with 1–2% isofluorane/air mixture, and body temperature was maintained using a heated air system (Air-Therm Heather, World Precision Instruments, Sarasota, FL). MR images were acquired prior to treatment initiation, daily during the first seven days for Study 1 and 3 days for Study 2 and every other day until the animals were sacrificed or became moribund.

MRI experiments consisted of two imaging sequences to measure tumor volume and tumor apparent diffusion coefficient (ADC). Delineation of tumor from healthy brain tissue was determined using a contrast-enhanced T1-weighted spin-echo images with the following parameters: Repetition time (TR)/echo time (TE) = 510/15 ms, field of view (FOV) = 20×20 mm^2^, matrix size = 128×128, slice thickness = 0.5 mm, 25 slices and 2 averages. Total acquisition time was 2 minutes and 12 seconds. Contrast-enhancement was performed by i.p. administration of 50 µl of 0.5 M gadolinium-DTPA (Magnevist, Bayer Healthcare Pharmaceuticals, Wayne, N.J) 5 minutes prior to image data acquisition. Tumor ADC maps were obtained from a diffusion-weighted spin-echo sequence, equipped with a navigator echo for motion correction and gradient waveforms sensitive to isotropic diffusion, with the following parameters: TR/TE = 2000/37 ms, FOV = 20×20 mm^2^, matrix size = 128×64, slice thickness = 0.5 mm, 25 slices, 2 averages, diffusion time = 40 ms, gradient pulse width = 10 ms and b-values (diffusion weighting) of 120 and 1200 s/mm^2^. Total acquisition time was 8 minutes and 32 seconds. DW-MRI scans were discontinued following day 15 post-treatment initiation.

### Image Reconstruction and Analysis

Volumes of interest (VOIs) were manually contoured along the enhancing rim of the tumors on the contrast-enhanced T1-weighted images for tumor volume measurements and determination of whole-tumor means of ADC. Tumor doubling times were determined over the first week of treatment by linearizing tumor volume measurements, calculating the slope (i.e. tumor growth rate) using a linear regression algorithm and dividing ln(2) to the rate. ADC maps were calculated from the two diffusion weightings (b-values) using the following equation:
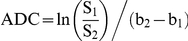
where S_1_ and S_2_ are the signal intensities at b-values b_1_ and b_2_, respectively, and ADC is the apparent diffusion coefficient obtained using b_1_ and b_2_. Voxels that exhibit insufficient signal, defined as <10*noise, in the low b-value image (b = 120 s/mm^2^) were excluded from the analysis. Subsequently, mean ADC values were calculated over the tumor volume. All image reconstruction and digital image analysis was accomplished using in-house programs developed in Matlab (The Mathworks, Natick, MA, USA).

### Protein study

Tumor tissue from untreated or treated animals was extracted, snap frozen and stored at −80 C. Lysis was performed using standard lysis buffer (Ripa) by homogenizing the tumor tissue. Western blotting for cleaved Caspase-3 was performed following standard procedures and the following antibodies: Caspase-3 (Cell Signaling), b-Actin (Abcam), Gapdh-HRP (Abcam).

### Histology

For each of the treatments, four animals from each group were sacrificed for histological analysis of the tumors at D2 and D7 (2 and 7 days after treatment initiation, respectively). Tissues were fixed in formalin, transferred to ethanol and embedded in paraffin. Tissue sections were stained with H&E (cell viability) and with cleaved Caspase-3 antibody (Cell Signaling) after antigen retrieval with Diva (Biocare) using the Avidin/Biotin complex system (Vectastain, Vector labs, Burlingame, CA) and disclosed with DAB Solution (Vector labs, Burlingame, CA).

### Statistics

Treatment efficacy on overall survival was assessed by log-rank test and displayed using Kaplan-Meier survival curves. Group comparisons of percent change in tumor volume and mean ADC were assessed at individual time points using a Analysis of Variance (ANOVA). All statistical computations were performed with a statistical software package (SPSS Software Products, Chicago, IL). Statistical significance was assessed at p<0.05.
